# Reporting of ethical approval and informed consent in clinical research published in leading nursing journals: a retrospective observational study

**DOI:** 10.1186/s12910-019-0431-5

**Published:** 2019-12-05

**Authors:** Yanni Wu, Michelle Howarth, Chunlan Zhou, Mingyu Hu, Weilian Cong

**Affiliations:** 1 Nanfang Hospital, Southern Medical University, Guangzhou, Guangdong People’s Republic of China; 2 The School of Health & Society, University of Salford, Greater Manchester, UK

**Keywords:** Clinical research, Informed consent, Ethical approval, Nursing journal, Research ethics

## Abstract

**Background:**

Ethical considerations play a prominent role in the protection of human subjects in clinical research. To date the disclosure of ethical protection in clinical research published in the international nursing journals has not been explored. Our research objective was to investigate the reporting of ethical approval and informed consent in clinical research published in leading international nursing journals.

**Methods:**

This is a retrospective observational study. All clinical research published in the five leading international nursing journals from the SCI *Journal Citation Reports* between 2015 and 2017 were retrieved to evaluate for evidence of ethical review.

**Results:**

A total of 2041 citations have been identified from the contents of all the five leading nursing journals that were published between 2015 and 2017. Out of these, 1284 clinical studies have been included and text relating to ethical review has been extracted. From these, most of prospective clinical studies (87.5%) discussed informed consent. Only half of those (52.9%) reported that written informed consent had been obtained; few (3.6%) reported oral consent, and few (6.8%) used other methods such as online consent or completion and return of data collection (such as surveys) to denote assent. Notably, 36.2% of those did not describe the method used to obtain informed consent and merely described that “*consent was obtained from participants or participants agreed to join in the research*”. Furthermore, whilst most of clinical studies (93.7%) mentioned ethical approval; 92.5% of those stated the name of ethical committee and interestingly, only 37.1% of those mentioned the ethical approval reference. The rates of reporting ethical approval were different between different study type, country, and whether financial support was received (all *P* < 0.05).

**Conclusion:**

The reporting of ethics in leading international nursing journals demonstrates progress, but improvement of the transparency and the standard of ethical reporting in nursing clinical research is required.

## Background

Given the importance of protecting human subjects in clinical research, there is now greater scrutiny of researchers to ensure that ethical principles have been met during the process [1, 2]. The World Medical Association issued the Declaration of Helsinki in 1964 and established the international ethical regulations for medical studies involving human subjects with subsequent updates [3]. The Declaration of Helsinki highlighted two aspects of ethical considerations: that all of the participants have the right to be informed about the study, by giving informed consent, and that an ethics committee approval should have got to ensure the appropriateness of design before initiating a research [4]. Furthermore, journals and publishers, have a responsibility to act as “gate-keepers”, and are obliged to scrutinize whether ethical approval of human research has been obtained priori to submission of papers [5]. This responsibility mirrors the requirement of the Declaration of Helsinki that “*Researchers, authors, sponsors, editors and publishers all have ethical obligations……Reports of research not in accordance with the principles of this Declaration should not be accepted for publication*” [4]. This duty of journals and publishers is supported by the International Committee of Medical Journal Editors (ICMJE) [6] and adopted by journal editors and publishers. Following this, the Nuremberg Code [7], the World Association of Medical Editors (WAME) [8], and the Committee of Publication Ethics (COPE) [9] established ethical principles to protect human right in medical research. For example, COPE stipulated that journals should provide guidance to support the reporting of ethical approval and informed consent when publishing human research.

However, the reporting of ethical considerations still less than ideal in human research though it is acknowledged that some progress has made in recent years. For example, Yank and Rennie [1] investigated the ethical protections of clinical trials published in top five medical journals including The Lancet, JAMA, BMJ, The New England Journal of Medicine, and Annals of Internal Medicine and found that 31 and 26% articles published before 1997 did not report ethical approval and informed consent, respectively. Furthermore, 18% of publications in these five journals after 1997 did not report ethical approval and informed consent. More recently, similar findings were identified by a range of authors, suggesting that this is a common challenge faced by journals [10–17]. For example, Schroter, Plowman [10] reviewed five general medical journals and reported that 47 and 31% of human research did not describe whether informed consent and ethical approval have obtained. More recently, the increasing concern on human rights protection has influenced this and ethical transgressions have improved. This was illustrated by Bridoux, Schwarz [18], who reported that 92.2% of surgical trials described informed consent and 87.7% stated ethical approval. However, publications that are not in accordance with the principles of ethical reporting remains common [19–22]. For example, Murphy, Nolan [20] identified that 42.9 and 49.9% of clinical research published in three leading European Otolaryngology periodicals did not report informed consent and ethical approval, respectively.

Nursing research has progressed rapidly during the last three decades and supported the development of efficient and high-quality care. This is observed in a number of nursing academic journals, increased volume of nursing research, and professors in nursing [23–25]. The growth in nursing research presents a range of challenges, not least because of the vulnerable groups that nursing research includes and the capability and capacity of nurses to conduct ethically sound research. Worryingly, it is acknowledged that many nurses receive inadequate education, often compounded with lack of ethical awareness and knowledge when conducting clinical research [26–29]. This was reflected in findings reported by Negarandeh and Gobady [30] who identified that 70.8% of nurses and midwives lack of education on ethical issues. These challenges within nursing research has raised concerns and the International Council of Nurses Code of Ethics for Nurses [31] has founded to regulate research ethics in the nursing profession. Therefore, it is essential to identify the extent to which ethical review is reported in nursing publications to both regulate and monitor ethics in nursing research involving human subjects.

Currently, the majority of ethics investigation have focused on reports in medical journals. In order to identify the extent to which ethical approval on human research is reported in nursing journals, we conducted a study to explore the ethical considerations among 12 Chinese top nursing journals. Our findings identified that only 51.8 and 25.9% of clinical trials reported informed consent and ethical approval, respectively [32]. The purposes of this study were to assess the rates of reporting of ethical considerations in five leading international nursing journals following the work of Yank and Rennie [1].

## Methods

### Study design and inclusion criteria

This is a retrospective observational study adhered to STROBE guidelines. All publications that reported clinical research in five high ranked nursing journals, according to the 2017 SCI *Journal Citation Reports’* impact factor between 2015 and 2017 were retrieved to evaluate for evidence of ethical review. These journals, with a high impact factor, were *International Journal of Nursing Studies* (3.755), *European Journal of Cardiovascular Nursing* (2.763), *Journal of Family Nursing* (2.537), *Nurse Education Today* (2.533), and *Birth-issues in Perinatal Care* (2.518). Publications were included if the following criteria were met: (1) Clinical research articles: as described by previous studies [10, 11, 13, 20], the sample frame selected included all original research articles involving human participants or human tissue. (2) Full-text articles. Supplement published studies, protocols, laboratory and animal studies, reviews, letters, editorials, discussion papers, erratum/corrigendum, commentaries, and news were excluded.

### Data extraction

Data were extracted between August 2017 and July 2018. The contents and full-text PDFs of the five top nursing journals between 2015 and 2017 were extracted and downloaded from the Wiley Online Library or the ScienceDirect through the university subscription. Two authors (W.C and M.H1) independently reviewed the articles published in the five nursing journals in keeping with the standardized eligibility criteria to ensure the accuracy and credibility of the process. All of the articles were identified and reviewed based on the contents of each issue of journal. The full text of each included article was carefully read and the results were recorded in a standardized data extraction form. Disagreements were resolved by consensus or the third person (Y.W). Following this, articles that did not report ethical review were checked again by the third person (Y.W).

The primary outcome of our study was to ascertain the rates of reporting informed consent and ethical approval. We included papers with any of the following types descriptions (with examples). (1) Informed consent – a. written informed consent obtained (“*the written informed consent was obtained from participants or the legally authorized representative*”), b. oral informed consent obtained (“*the oral/verbal consent was obtained from participants*”), c. other consent type (has been categorized as “*the participants’ consent was indicated by the completion and returning of the questionnaire*”, “*consent was obtained through providing information and finishing consent section or a consent form within the online portal*”, and “*the informed consent was indicated by participants take part in evaluations*”), d. the consent type not reported (only described that “*consent was obtained from participants or participants agreed to join in the research*”, but did not indicate the way to get the consent), and e. consent was waived or not required (“*informed consent was exempted or not required due to the policy or the law of the government or the type of the research*”). The dates on informed consent in the study were collected on prospective studies only [20]. (2) Ethical approval – we checked to determine if the study reported that it was approved by the ethics committee in the hospital or other institutions before undertaking the research. We also examined whether the name of the ethical committee and ethical approval reference number was reported.

The secondary outcomes of our study included the rates of reporting other details related to ethical approval and informed consent including ethical statement. For example, whether the author declared that the research conformed to the Declaration of Helsinki, and if the research participants had been told that they have the right to withdraw from the study at any time without reprisal. There was excellent agreement on the two primary outcome measures between two authors (W.C and M.H1) (reporting of informed consent and ethical approval) (*k* > 0.95 for all).

We collated additional information that included the study type, funding sources, and nationality of publishing institution to enable subgroup analysis. Firstly, the type of research was categorized as either prospective or retrospective based on prior studies [11, 13, 15]. Following this, the prospective studies were divided into randomized controlled trial (RCT), nonrandomized trial, observational study (including audit, surveys, quality assurance activities, prospective cohort study, and qualitative study), single-arm, specimen, and mixed methods study. The retrospective studies were divided into specimen, chart review, and database analysis. Secondly, data were gathered on whether research received financial support following the work of Yank and Reinnie [1], regardless of the type of funding. Finally, given the different clinical ethical dilemmas in different countries [33], we identified the nationality of publishing institution of each study, defined as the country of the research conducted institution.

### Data analysis

Data was analyzed using SPSS 20.0 software (IBM, USA). The rates of informed consent and ethical approval between year, study types, funding sources, and different countries were compared by use of Chi-square tests or Fisher’s exact tests (where cell size was less than 5). All *P* values were two-sided, and a value of *P <* 0.05 was indicated significant.

## Results

### Study selection

**Fig. 1:**
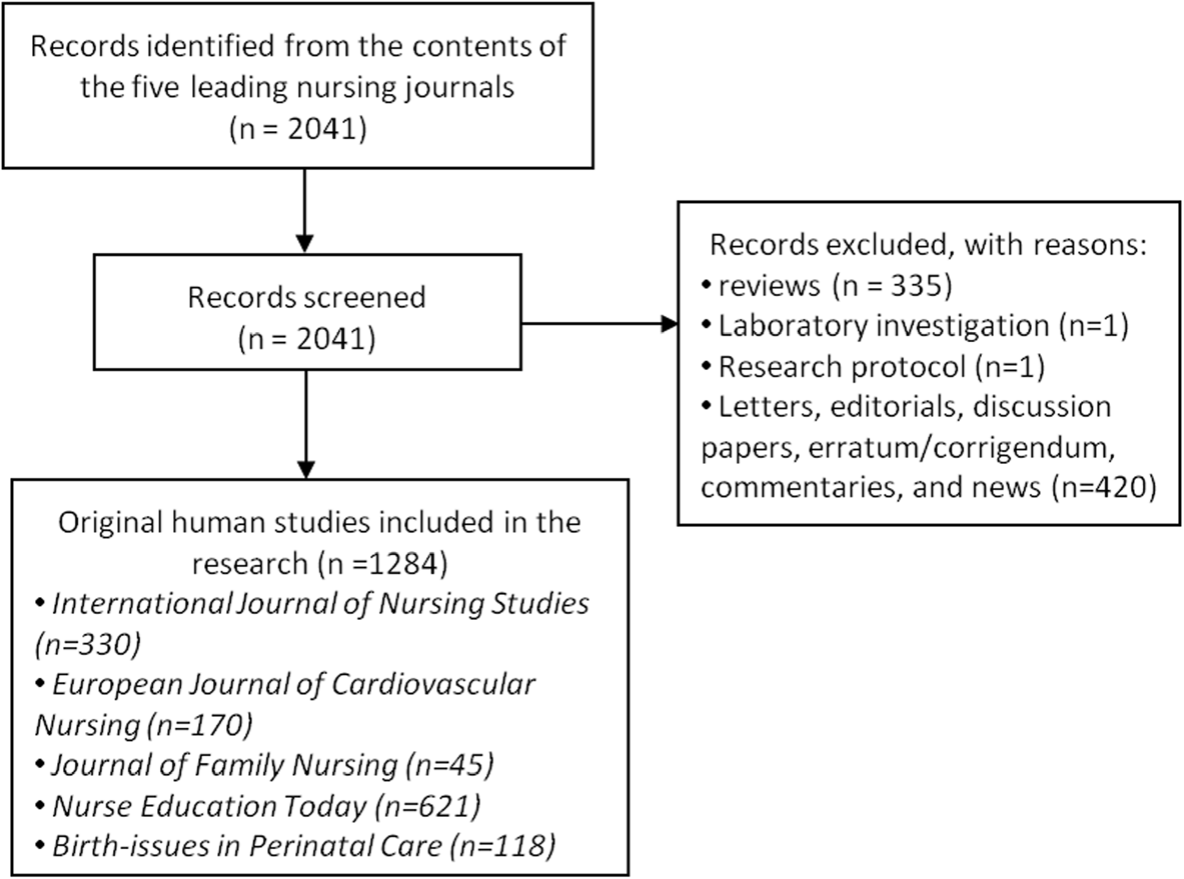
PRISMA Flow Diagram

A total of 2041 citations were identified from the contents of all the five leading nursing journals published between 2015 and 2017. From these, 757 were screened out using the data extraction process. These were: 335 reviews, one laboratory investigation, one research protocol, and 420 other types (letters, editorials, discussion papers, erratum/corrigendum, commentaries, and news). In total, 1284 clinical papers were included to extract the data of ethical review (see Fig. 1).

### Informed consent

Of the 1284 clinical studies, 99 were retrospective studies and 1185 were prospective studies. Of the 1185 prospective studies, 1037 (87.5%) mentioned informed consent. However, only 549 (52.9%) of those reported that written informed consent had been obtained, and only 37 (3.6%) of these had reported oral consent. Interestingly, 70 (6.8%) of studies had used other ways to indicate participants’ consent, such as ‘assent’ through the completion and return of the questionnaire in surveys, or finishing online consent section, or implied by participants attend research evaluations. Notably, a small number 375 (36.2%) described that “*consent was obtained from participants or participants agreed to join in the research*” but did not elaborate on the methods used to gain consent. Furthermore, six (0.5%) studies stated that the informed consent from participants were waived or not required due to the policy or the law of the government or the type of the research (Table 1). In addition, the rates of reporting informed consent between different publication years and evidence of research funding demonstrated no statistical significance (all *P* > 0.05) but variances were observed between different country (*P* < 0.001) (Table 2).

**Table 1:** Ethical review of studies published in leading nursing journals between 2015 and 2017

Journal	Number of studies (*N*, %)			Informed consent in prospective studies (*N*, %)						Ethical approval (*N*, %)	Ethical statement (*N*, %)
	Total	Prospective studies	Retrospective studies	Total	Written consent obtained	Oral consent obtained	Other consent type	The consent type not reported	Waived or not required		
International Journal of Nursing Studies	330	300 (90.9)	30 (9.1)	259 (86.3)	159 (61.4)	15 (5.8)	4 (1.5)	76 (29.4)	5 (1.9)	324 (98.2)	38 (11.5)
European Journal of Cardiovascular Nursing	170	160 (94.1)	10 (5.9)	151 (88.8)	104 (68.9)	1 (0.7)	3 (2.0)	43 (28.4)	0	164 (96.5)	107 (62.9)
Journal of Family Nursing	45	45 (100)	0	41 (91.1)	32 (78.1)	1 (2.4)	0	8 (19.5)	0	43 (95.6)	4 (8.9)
Nurse Education Today	621	611 (98.4)	10 (1.6)	525 (85.9)	217 (41.3)	16 (3.1)	62 (11.8)	229 (43.6)	1 (0.2)	567 (91.3)	32 (51.5)
Birth-issues in Perinatal Care	118	69 (58.5)	49 (41.5)	61 (88.4)	37 (60.7)	4 ((6.6)	1 (1.6)	19 (31.1)	0	105 (89.0)	0
Total	1284	1185 (92.3)	99 (7.7)	1037 (87.5)	549 (52.9)	37 (3.6)	70 (6.8)	375 (36.2)	6 (0.5)	1203 (93.7)	181 (14.1)

**Table 2:** Reporting of informed consent and ethical approval in clinical research in leading nursing journals between 2015 and 2017

Items	Number of clinical researches	Number of Prospective studies	*N* (%)	
			Informed consent reported	Ethical approval reported
Year				
2015	398	364 (91.5)	328 (90.1)	376 (94.5)
2016	510	477 (93.5)	411 (86.2)	477 (93.5)
2017	376	344 (91.5)	298 (86.6)	350 (93.1)
*X*^*2*^			0.462	0.045
*P* value			0.794	0.978
Study type				
Prospective studies	1185	–	1037 (87.5)	1121 (94.6)
Randomized controlled trial	122	–	113 (92.6)	119 (97.5)
Nonrandomized trial	62	–	52 (83.9)	61 (98.4)
Observational study	835	–	734 (87.9)	792 (94.9)
Single-arm	66	–	52 (78.8)	55 (83.3)
Specimen	0	–	0	0
Mixed methods study	100	–	86 (86.0)	94 (94.0)
Retrospective studies	99	–	–	82 (82.8)
Specimen	0	–	–	0
Chart review	21	–	–	20 (95.2)
Database analysis	78	–	–	62 (79.5)
*P* value			–	< 0.001
Mentioning financial support				
Yes	702	662 (94.3)	587 (88.7)	668 (95.2)
No	582	523 (89.9)	450 (86.0)	535 (91.9)
*P* value			0.185	0.021
The country of conducting the research				
US	186	155 (83.3)	127 (81.9)	175 (94.1)
Australia	148	137 (92.6)	118 (86.1)	147 (99.3)
UK	144	136 (94.4)	111 (81.6)	138 (95.8)
China	124	119 (96.0)	107 (90.0)	118 (95.2)
Sweden	69	63 (91.3)	59 (93.7)	66 (95.7)
≥ 2 countries	63	59 (93.7)	48 (81.4)	52 (82.5)
Canada	60	55 (91.7)	51 (92.7)	58 (96.7)
Netherlands	47	44 (93.6)	39 (88.6)	42 (89.4)
Spain	43	40 (93.0)	35 (87.5)	31 (72.1)
Korea	43	41 (95.3)	39 (95.1)	43 (100)
Turkey	33	33 (100)	33 (100)	28 (84.9)
Norway	25	23 (92.0)	22 (95.7)	24 (96.0)
Italy	25	23 (92.0)	21 (91.3)	23 (92.0)
Japan	19	16 (84.2)	15 (93.8)	19 (100)
Finland	17	16 (94.1)	14 (87.5)	15 (88.2)
Ireland	17	16 (94.1)	12 (75.0)	13 (76.5)
Singapore	17	17 (100)	16 (94.1)	16 (94.1)
Israel	16	12 (75.0)	9 (75.0)	16 (100)
Germany	15	14 (93.3)	11 (78.6)	13 (86.7)
Denmark	14	14 (100)	14 (100)	13 (92.9)
Belgium	13	13 (100)	12 (92.3)	13 (100)
Iran	13	13 (100)	12 (92.3)	13 (100)
Jordan	11	11 (100)	10 (90.1)	10 (90.1)
Brazil	11	10 (90.1)	9 (90.0)	11 (100)
France	9	7 (77.8)	5 (71.4)	8 (88.9)
New Zealand	8	6 (75.0)	4 (66.7)	8 (100)
Switzerland	8	8 (100)	8 (100)	7 (87.5)
Thailand	7	7 (100)	6 (85.7)	7 (100)
South Africa	6	6 (100)	6 (100)	6 (100)
Greece	5	5 (100)	4 (80.0)	5 (100)
Poland	5	5 (100)	5 (100)	5 (100)
Portugal	5	5 (100)	5 (100)	5 (100)
Iceland	4	3 (75.0)	3 (100)	3 (75.0)
India	4	4 (100)	3 (75.0)	4 (100)
Indonesia	4	4 (100)	3 (75.0)	4 (100)
Malaysia	4	4 (100)	4 (100)	4 (100)
Lebanon	4	4 (100)	4 (100)	4 (100)
Philippines	4	4 (100)	4 (100)	4 (100)
Saudi Arabia	4	4 (100)	2 (50.0)	3 (75.0)
Croatia	3	3 (100)	2 (66.7)	3 (100)
Afghanistan	2	2 (100)	2 (100)	2 (100)
Africa	2	2 (100)	2 (100)	2 (100)
Chile	2	1 (50.0)	1 (100)	2 (100)
Mexico	2	2 (100)	2 (100)	2 (100)
Nepal	2	2 (100)	2 (100)	2 (100)
Oman	2	2 (100)	2 (100)	2 (100)
Brunei	1	1 (100)	1 (100)	1 (100)
Dutch	1	1 (100)	1 (100)	1 (100)
Egypt	1	1 (100)	1 (100)	1 (100)
Ethiopia	1	1 (100)	1 (100)	1 (100)
Florida	1	1 (100)	1 (100)	1 (100)
Lithuania	1	1 (100)	1 (100)	1 (100)
Madagascar	1	1 (100)	1 (100)	0
Malawi	1	1 (100)	1 (100)	1 (100)
Nigeria	1	1 (100)	1 (100)	1 (100)
Pakistan	1	1 (100)	1 (100)	1 (100)
Palestine	1	1 (100)	1 (100)	1 (100)
Qatar	1	1 (100)	1 (100)	1 (100)
Serbia	1	1 (100)	1 (100)	1 (100)
Slovenia	1	1 (100)	0	1 (100)
Srilanka	1	1 (100)	1 (100)	1 (100)
*P* value			< 0.001	< 0.001

### Ethical approval

Our results indicate that 1203 (93.7%) of 1284 clinical studies reported ethical approval in the main text of the paper. Of the 1203 studies, 1144 (95.1%) reported that ethics committee approval was obtained before the study was undertaken and 59 (4.9%) of studies stated that the ethical approval for the study was not required or waived under the local or national laws. Notably, of the 1144 studies that reported ethical approval had been obtained, a larger number, 1058 (92.5%) of studies stated the name of ethical committee, however, only 424 (37.1%) of studies included the ethical approval reference number. A small number, 181 (14.1%) of studies stated that ethical considerations of the research conformed to the Declaration of Helsinki (Table 1). Furthermore, no statistically significant differences were found between 2015 and 2017 in relation to the rates of reporting ethical approval (*P* > 0.05). Notably, the rates of reporting ethical approval were different between different study type, country, and whether mentioning financial support (all *P* < 0.05). The rates of reporting ethical approval in prospective studies was much higher than retrospective studies (94.6% vs 82.8%). Moreover, the reporting of ethical approval in the studies that had financial support, was much higher than those who did not receive funding (95.2% vs 91.9%) (Table 2).

## Discussion

Our findings revealed a relative progression in the reporting of ethics as compared with prior studies such as Yank and Rennie [1], Schroter, Plowman [10], Pitak-Arnnop, Sader [14], and Fitzgerald [19]. For example, we identified that 87.5% of prospective clinical studies reported informed consent and 93.7% of clinical studies stated that ethical approval had been obtained. This is similar to Bridoux, Schwarz [18] findings that reported 87.7 and 92.2% of surgical trials stated ethical approval and informed consent, respectively. However, the reporting rates of ethics were much higher than most of other studies. For example, informed consent and ethical approval were reported in 53 and 69% in five general medical journals, 68.5 and 71% in six leading anesthesia journals, 36 and 39.3% in four major orthodontic journals, 57.1 and 50.1% in three leading European Otolaryngology journals, as well as 16 and 54% in three paediatric surgical journals, respectively [10, 11, 19, 20, 22]. Furthermore, the reporting of ethical considerations in the five leading international journals was more frequent than the twelve top Chinese nursing journals we previously investigated in 2017. These earlier findings highlighted that only 51.8 and 25.9% of clinical trials stated informed consent and ethical approval, respectively [32]. This more recent increase may have been influenced though the rapid development of medical technology which has enriched the content of nursing clinical research. However, it is acknowledged that China’s higher nursing education developed slowly because of civil wars and external invasion until 1949 and still need more efforts to improve the knowledge and awareness of ethics among nursing researcher [34].

Importantly, our findings indicated that the reporting of ethical approval in leading international nursing journals is less than ideal and work is needed to develop a standardized approach. Whilst our research illustrates a welcome progression, equally, the reporting of ethical approval is now recognized by leading journals as an essential pre-publication requirement. However, the detail of *what* is reported needs to be developed to enable readers and editors to understand that the reporting of consent was more than just ‘consent’ and that it was truly informed, thus reflecting the autonomous rights of the research participants. Merely stating fact that ‘informed consent’ was obtained, does not necessarily mean that consent was actually informed [1]. Signing a consent form or explicitly negotiated verbal consent presents two traditional methods in which participants’ informed consent is obtained [3]. With the rapid development of network techniques, using electronic methods to obtain informed consent is now considered to be a convenient as compared to other ways especially in some online questionnaire survey research. Furthermore, based on the 375 studies that did not describe the way to get the consent, determining whether the ethical considerations reported were implemented as challenging. Moreover, the reason for exemption should be declared by the authors which should include a rationale for absence of informed consent and/or ethical approval. For example, lack of informed consent maybe as a result of fully anonymised samples or legal reasons [15]. In our study, only 59 (4.9%) of studies stated that the ethical approval for the study was not required or waived under the local or national laws. Of the studies that have been identified as not reporting ethical considerations, we are unable to report whether the ethical protections of these studies were deemed unnecessary or if the researcher did not consider it. Therefore, the reporting of ethical protection of clinical research in leading nursing journals needs to be transparent and standardized.

Furthermore, our study identified progression in the reporting of ethical approval, however, only 37.1% of studies mentioned the ethical approval reference number. Although the ethical principles of the Declaration of Helsinki and the ICMJE do not stipulate that authors report the name of ethics committee and the approval number in the text, it has been recommended by many researchers [1, 20, 35]. Similar reporting expectations are included in the author instructions in numerous medical journals such as the *BMJ*, the *European Journal of Cardiovascular Nursing*, and the *Journal of Family Nursing*. This conflict in reporting of ethical committees and references numbers could be explained by the following two reasons. Firstly, the inclusion of the ethics committee details and reference helps to regulate ethical statement and to make sure that the ethical approval is authentic. Secondly, there is strong evidence to show the different effectiveness and practice between different local committees, stating the name of ethics committee could also help readers or others make decisions on the ethical protections of the study [36, 37]. In addition, our study, only five clinical studies stated the organizational approval to conduct the research including the approval from the dean’s office, the school management, and the head of the department. Though this may be due to the organization policy, this approach does not comply with the principles of the Declaration of Helsinki which states that the research should be approved by an independent ethics committee. Therefore, the statement of the ethics committee approval in clinical studies still need to be standardized and improved.

It was surprising that there was no difference between publication year on ethical considerations in leading international nursing journals, which is contrary to the results on twelve Chinese nursing journals demonstrated a sharp improvement previously identified between 2013 and 2016 [32]. This may have been influenced by recent higher education institution, the ethics committee, and the nursing journals efforts in China in recent years.

Furthermore, our research demonstrated that the rates of reporting ethical approval in prospective studies was much higher than retrospective studies (94.6% vs 82.8%). Specifically, RCTs showed a relatively high reporting rates whereas the study type of database and single-arm need more attention to improve the reporting of ethical considerations. This is supported by Block [13] who found that the RCTs showed a high proportion in reporting of ethical approval. Most of researchers obtained ethics committee approval for RCTs and were not realized that approval still required for studies that do not enroll human participants such as retrospective studies on chart or database review [15]. Moreover, although we only retrieved the rates of reporting informed consent in prospective studies in line with prior studies [13, 20], we are aware that neither retrospective studies published in the five leading international nursing journals stated the informed consent. The Declaration of Helsinki states that if informed consent for medical research using existing human data or material is impracticable to obtain, the research could be done after approval of an ethics committee. Furthermore, some researchers also stated that the informed consent is not required to the retrospective studies because this kind of study meet the criteria that “*the research involves no more than minimal risk to the subjects*” [13].

## Conclusion

In summary, nursing journals assume an extremely important social, moral and ethical responsibility to improve and regulate the reporting of ethical considerations in clinical research. The reporting of ethics in the five leading international nursing journals showed some progress, but effort is still required to standardize the transparency and detail of ethical reporting.

## Data Availability

The datasets used and/or analyzed during the current study are available from the corresponding author on reasonable request.

## Abbreviations

COPE: The Committee of Publication Ethics
ICMJE: The International Committee of Medical Journal Editors
RCTs: Randomized controlled trials
WAME: The World Association of Medical Editors
